# Eumelanin and pheomelanin pigmentation in mollusc shells may be less common than expected: insights from mass spectrometry

**DOI:** 10.1186/s12983-019-0346-5

**Published:** 2019-12-23

**Authors:** Susanne Affenzeller, Klaus Wolkenstein, Holm Frauendorf, Daniel J. Jackson

**Affiliations:** 1 Department of Geobiology, Georg-August University of Göttingen, Goldschmidtstr. 3, 37077 Göttingen, Germany; 2 Institute of Organic & Biomolecular Chemistry, Georg-August University of Göttingen, Tammannstr. 2, 37077 Göttingen, Germany

**Keywords:** Eumelanin, Pheomelanin, Mollusc, Shell, Pigment, Liquid chromatography–mass spectrometry, Colour pattern

## Abstract

**Background:**

The geometric patterns that adorn the shells of many phylogenetically disparate molluscan species are comprised of pigments that span the visible spectrum. Although early chemical studies implicated melanin as a commonly employed pigment, surprisingly little evidence generated with more recent and sensitive techniques exists to support these observations.

**Results:**

Here we present the first mass spectrometric investigations for the presence of eumelanin and pheomelanin in 13 different molluscan species from three conchiferan classes: Bivalvia, Cephalopoda and Gastropoda. In the bivalve *Mytilus edulis* we demonstrate that eumelanin mainly occurs in the outermost, non-mineralised and highly pigmented layer of the shell (often referred to as the periostracum). We also identified eumelanin in the shells of the cephalopod *Nautilus pompilius* and the marine gastropods *Clanculus pharaonius* and *Steromphala adriatica*. In the terrestrial gastropod *Cepaea nemoralis* we verify the presence of pheomelanin in a mollusc shell for the first time. Surprisingly, in a large number of brown/black coloured shells we did not find any evidence for either type of melanin.

**Conclusions:**

We recommend methods such as high-performance liquid chromatography with mass spectrometric detection for the analysis of complex biological samples to avoid potential false-positive identification of melanin. Our results imply that many molluscan species employ as yet unidentified pigments to pattern their shells. This has implications for our understanding of how molluscs evolved the ability to pigment and pattern their shells, and for the identification of the molecular mechanisms that regulate these processes.

## Background

Shell bearing molluscs (Conchifera Gegenbauer, 1878) constitute one of the most abundant and diverse groups of extant and extinct life [1–4]. The colouration and patterning of the molluscan shell and associated biominerals (e.g. pearls) have fascinated human cultures since prehistoric times [5–10]. The pigmentation of these structures holds not only aesthetic beauty, but can also dictate their commercial value [11–13]. The evolution of the molecular mechanisms that both synthesise and deposit these pigments, and the way this is achieved in such coordinated and visually attractive patterns is of great interest to many fields of evolution, ecology and cellular biology. It is therefore surprising that these pigments (which range from blue, red and yellow to monochromatic brown/black and white) are not well characterised [14]. Early chemical studies based on chromatographic properties and UV–visible spectra of pigments carried out by Comfort [7, 15–19] and Helmcke [20] identified the presence of different classes of organic pigments, including tetrapyrroles and melanins. More recent studies have shown that tetrapyrroles (porphyrins and biliverdins) and carotenoids are present in colourful mollusc shells [14, 21–26], with melanins being associated with dark purple, brown and black shell patterns most often [7, 19, 21, 27, 28]. For example, black/brown eumelanin has been linked to the dark colouration of pearls [11, 29–32]. Despite the common association of melanin with dark colours in mollusc shells, very few studies have used extensive analytical methods to support its presence. Evidence of eumelanin was reported recently in the shells of *Clanculus* (Gastropoda) which bear black dots (via high-performance liquid chromatography with UV detection (HPLC–UV)), and in the bivalves *Mizuhopecten yessoensis* (HPLC–UV), *Pteria penguin* (HPLC with mass spectrometric detection (HPLC–MS)) and *Crassostrea gigas* (infrared absorption spectra) [21, 30, 31, 33]. However, analysing mollusc shells for melanins is challenging due to the presence of complex organic matrices which generate high backgrounds (see chromatograms in the above publications). Moreover, melanins are complex macromolecules that are generally very difficult to analyse [34, 35]. Finally, to complicate matters further, the term ‘melanin’ has been used in the literature as an umbrella term in reference to black/brown and reddish to yellow pigments that are non-soluble and very stable. Here we define melanin as the product of enzymatic oxidative polymerisation of DOPA (L-3,4-dihydroxyphenylalanine) subunits. While different methods for the characterization of melanins in biological samples have been reported, for example Raman spectroscopy [23, 29, 36], electron spin resonance spectroscopy [37, 38] and pyrolysis–gas chromatography–mass spectrometry [38, 39], these all provide only limited structural information. Currently, only one identification method is well established and accepted in melanin research [40, 41], namely the analysis of characteristic oxidation products following alkaline oxidation of the melanin polymers [41, 42]. After alkaline oxidation the products PDCA (pyrrole-2,3-dicarboxylic acid) and PTCA (pyrrole-2,3,5-tricarboxylic acid) for eumelanin and TDCA (thiazole-4,5-dicarboxylic acid) and TTCA (thiazole-2,4,5-tricarboxylic acid) for pheomelanin can be analysed with HPLC–UV [38, 41, 43]. However, distinguishing these specific melanin markers from background signals resulting from the oxidation of proteins and other compounds without mass information is challenging. We have recently demonstrated that a sample preparation and clean-up step after alkaline oxidation, followed by HPLC–UV–MS permits the unequivocal detection of even trace amount of melanins in mollusc shells [44].

**Fig. 1:**
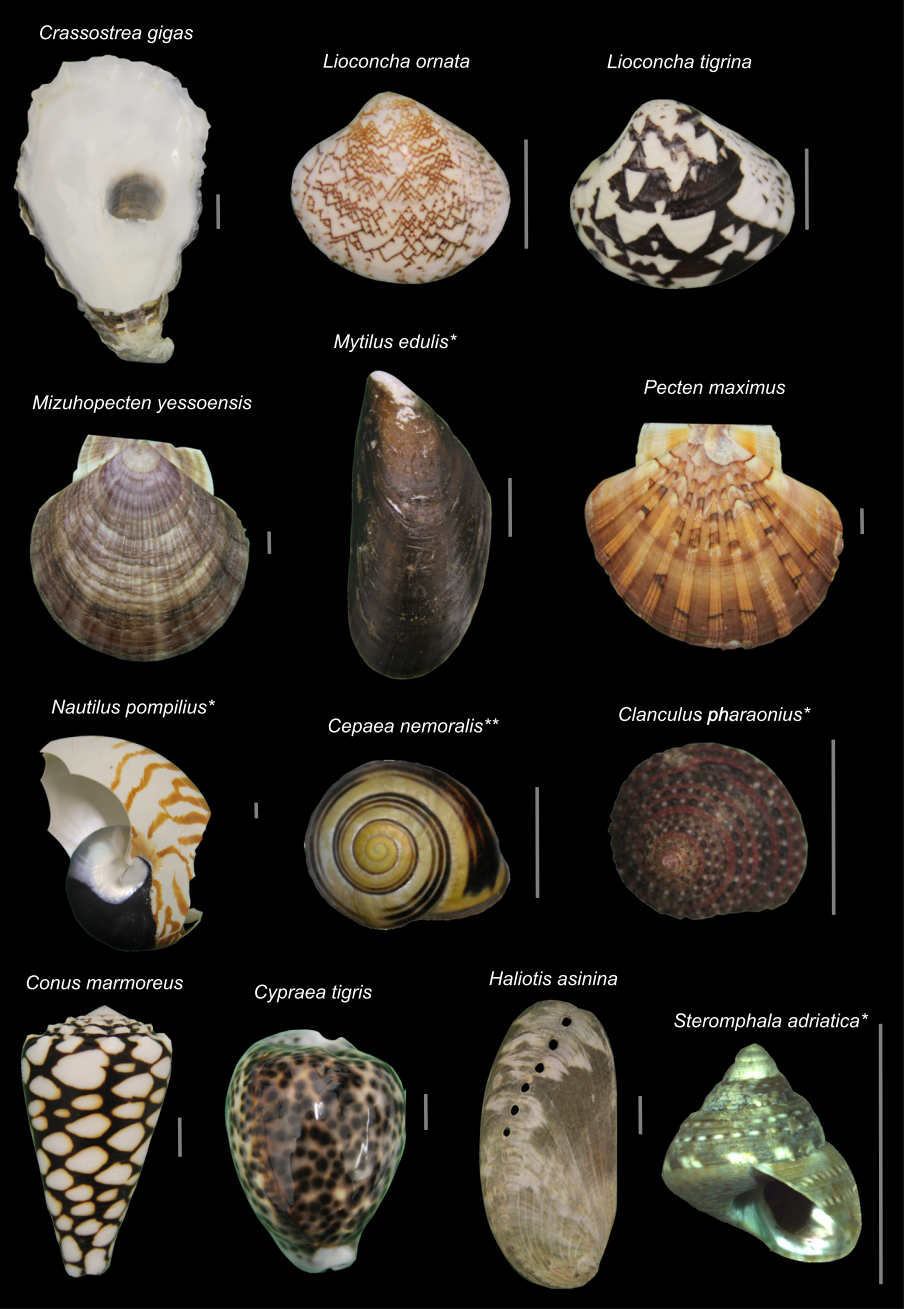
Pigmented shell samples investigated for eumelanin and pheomelanin colouration. Evidence for eumelanin is marked with an asterix, evidence for both eumelanin and pheomelanin is marked with two asterix. (Scale bars are 1 cm)

Here we investigate the presence of eumelanin and pheomelanin pigmentation in 13 different species of shell bearing molluscs (Table 1) using alkaline oxidation followed by HPLC–UV–MS. All of these species display prominent patterns on their shells with colours ranging from yellow, light brown, orange and red to dark brown and black (Fig. 1). A number of these species are of significant commercial or cultural value (*Mytilus edulis, Pecten maximus, Cypraea tigris*, *Haliotis asinina*), while others (*Crassostrea gigas*, *Mizuhopecten yessoensis*, *Cepaea nemoralis*, *Clanculus pharaonius*) were chosen due to previous reports of melanic pigmentation [19–21, 28, 30, 33].

**Table 1:** The shells of 13 different mollusc species investigated in this study

Class	Species	Eumelanin	Pheomelanin	Previous reports of eumelanin and methods used
Bivalvia	*C. gigas*^*1*^	–	–	[30] UV and IR spectroscopy
Bivalvia	*L. ornata*^*3*^	–	–	–
Bivalvia	*L. tigrina*^*3*^	–	–	–
Bivalvia	*M. yessoensis*^*3*^	–	–	[33] UV spectrophotometry and HPLC–UV
Bivalvia	*M. edulis*^*2*^	+	–	–
Bivalvia	*P. maximus*^*3*^	–	–	–
Cephalopoda	*N. pompilius*^*4*^	+	–	–
Gastropoda	*C. nemoralis*^*5*^	+	+	[7, 20] solubility tests
Gastropoda	*C. pharaonius*^*6*^	~ + ^*^	–	[21] HPLC–UV
Gastropoda	*C. marmoreus*^*3*^	–	–	–
Gastropoda	*C. tigris*^*3*^	–	–	–
Gastropoda	*H. asinina*^*7*^	–	–	–
Gastropoda	*S. adriatica*^*8*^	+	–	–

A “+” signifies the presence of oxidation products of eumelanin or pheomelanin in our HPLC–MS analyses, a “- “signifies we could not detect any melanin oxidation products. ^1^purchased from food market (Vienna, March 2018), ^2^purchased from grocery store (Göttingen, March 2018), ^3^purchased from Conchology Inc. (www.conchology.be), ^4^Donation from M. Hundertmark private collection, ^5^collected at botanical gardens of Georg-August University of Göttingen (September 2017), ^6^Donation from Natural History Museum Vienna (1 sample) and purchased from Schnecken und Muscheln (www.schnecken-und-muscheln.de) (2 samples), ^7^Donation from D. J. Jackson private collection, ^8^collected from Jackson group aquarium (December 2017–March 2018). ^*^Eumelanin was detected in one out of three samples

## Results and discussion

### Evidence of melanins in mollusc shells

This study currently represents the largest screen for melanins in molluscan shells using mass spectrometry. Using our newly developed HPLC–UV–MS method [44] which in contrast to HPLC–UV provides high selectivity, we unequivocally demonstrate the presence of eumelanin in five mollusc species belonging to three major clades of Conchifera. In addition, we found the first conclusive evidence of pheomelanin in a terrestrial gastropod (*Cepaea nemoralis*) known for its colour and banding polymorphism [45]. However, we also demonstrate that previous reports of eumelanin in two species (*Crassostrea gigas* and *Mizuhopecten yessoensis*) were possibly technical artefacts (see below), and that for a total of eight of the 13 species we investigated, which have brown/black pigmented patterns on their shells, we could find no evidence of melanin in their shells.

**Fig. 2:**
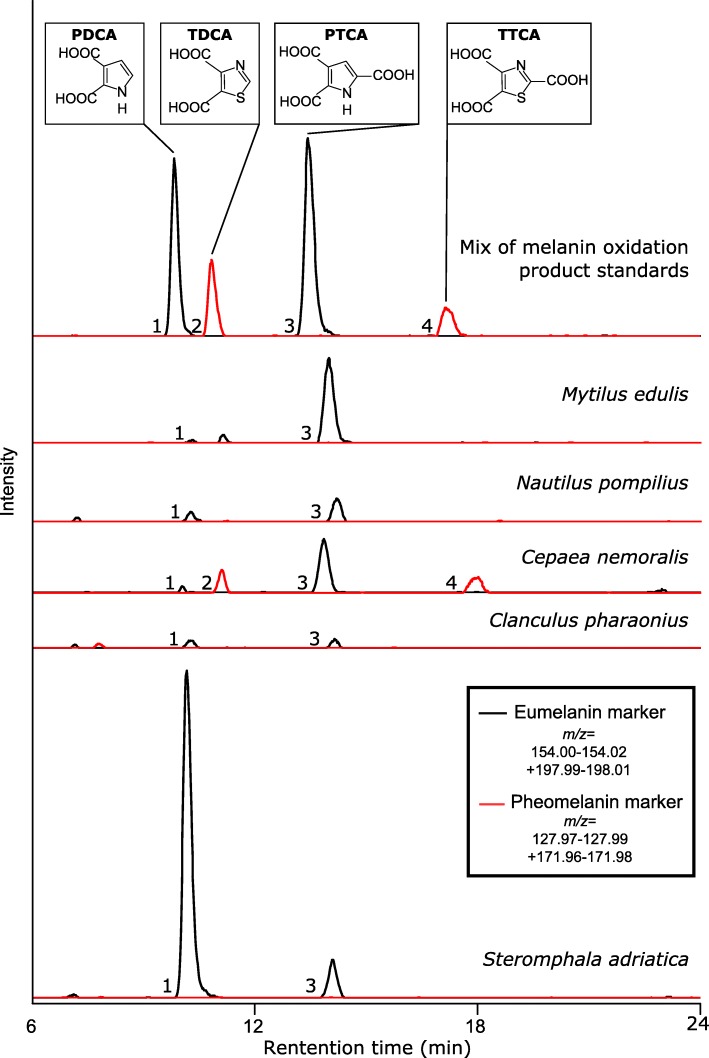
Extracted ion chromatograms (negative-ion mode) for eumelanin (black) and pheomelanin (red) specific oxidation products. Evidence for melanin in shell pigmentation was found in one bivalve (*Mytilus edulis*), one cephalopod (*Nautilus pompilius*) and three gastropods (*Cepaea nemoralis, Clanculus pharaonius*, *Steromphala adriatica*). All other species investigated showed no detectable signal for melanin oxidation products (chromatograms not pictured here). Note that sample extract of *Steromphala adriatica* shows degradation of PTCA to PDCA following sample storage (− 20 °C for 32 weeks)

**Fig. 3:**
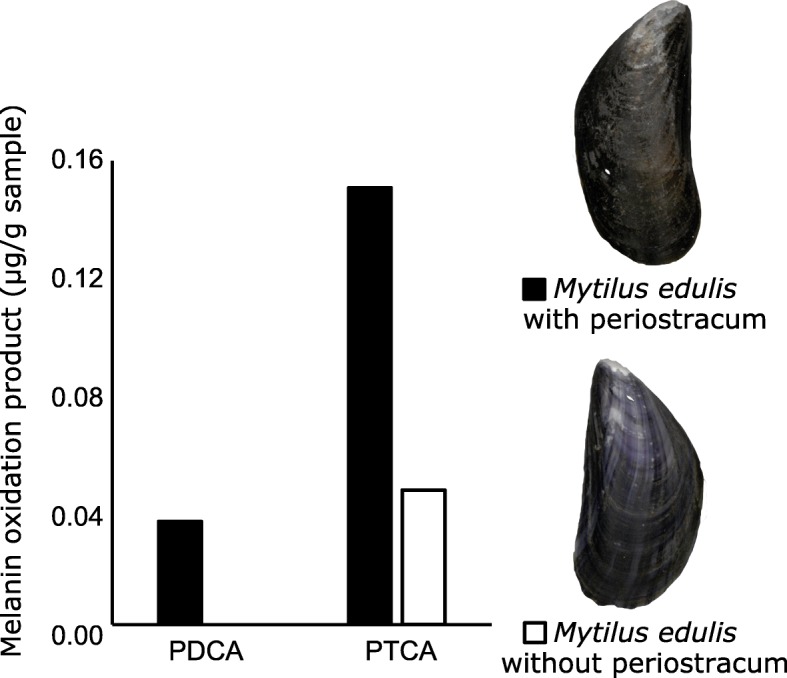
To test the predominance of eumelanin in different shell layers of *Mytilus edulis* the outermost brown periostracum was removed from one shell valve while the other valve was measured with intact periostracum. Eumelanin oxidation products PDCA and PTCA were quantitated by HPLC with UV detection with external calibration and measurements were normalized to initial sample weight

In the oxidised sample of *Mytilus edulis* we detected the characteristic eumelanin oxidation products PDCA and PTCA as revealed by ion chromatograms of their deprotonated and decarboxylated molecules (PDCA: *m*/*z* 154.01 [M–H]^−^, PTCA: *m*/*z* 198.00 [M–H]^−^ and *m*/*z* 154.01 [M–COOH]^−^) (Fig. 2). *Mytilus edulis* is a commercially relevant food source and is readily available, however surprisingly little literature is available on its pigmentation. The measurements we present here and in Affenzeller et al. [44] corroborate the findings of Waite and Andersen [46] who found that DOPA decreases along the shell growth axis, which is likely due to DOPA being polymerized to eumelanin [46, 47]. To further investigate Waite and Andersens’ [46] observations on the colour differences between the outermost brown periostracal layer and the underlying blue or purple banded calcified shell, we removed the periostracum from one shell valve and compared the amounts of eumelanin markers to the matched intact valve. This analysis provides the first evidence of the periostracum being the main source of eumelanic pigmentation in *Mytilus edulis* with the intact valve yielding approximately four times more PTCA than the valve without periostracum (Fig. 3 and Table 2).

**Table 2:** Amounts of melanin markers in molluscan shells (μg per g of sample)

Sample	Eumelanin markers		Pheomelanin markers	
	PDCA (μg/g)	PTCA (μg/g)	TDCA (μg/g)	TTCA (μg/g)
*M. edulis*				
with periostracum	0.04	0.15	n.d.	n.d.
without periostracum	< 0.01	0.05	n.d.	n.d.
*N. pompilius*	0.01	0.03	n.d.	n.d.
*C. nemoralis*	0.02	0.07	0.94	0.33
*C. pharaonius*	0.11	0.02	n.d.	n.d.
*S. adriatica*	0.50	0.06	n.d.	n.d.

Values are not corrected for recovery. Recoveries following solid-phase extraction for PDCA, PTCA, TDCA and TTCA were 82, 76, 95 and 67%, respectively [44]. Values for *N. pompilius* and *S. adriatica* were determined following storage of sample extracts (32 weeks, − 20 °C). HPLC–MS data obtained prior to and following sample storage indicated that in the *S. adriatica* sample originally PTCA was the predominant marker, suggesting degradation of PTCA to PDCA. n.d., not detected

The eumelanin markers PDCA and PTCA were also detected in oxidised *Nautilus pompilius* shell fragments with brown flame colouration (Figs. 1, 2 and Table 2). It is well-known that cephalopods use eumelanin in their ink [38, 43]. We show here that the ability to produce melanin is not only used as a defensive mechanism, but also contributes to external shell colouration in *Nautilus pompilius*. This finding might be of interest to palaeontologists working on shell bearing Cephalopoda, as colour patterns can be observed in fossilized specimens [48]. Our method might allow for the chemical analyses of melanin in these fossilized shells.

Within the gastropods we found melanin markers in the oxidised samples of three species: *Cepaea nemoralis*, *Clanculus pharaonius* and *Steromphala adriatica* (Table 1, Table 2). For the terrestrial gastropod *Cepaea nemoralis* we found mass spectrometric evidence for all four melanin oxidation products (Fig. 2 and Table 2), the characteristic pheomelanin markers TDCA and TTCA revealed by the ion chromatograms of the deprotonated molecule and ions resulting from the loss of one and two carboxyl groups (TDCA: *m*/*z* 171.97 [M–H]^−^ and *m*/*z* 127.98 [M–COOH]^−^, TTCA: *m*/*z* 171.97 [M–COOH]^−^ and *m*/*z* 127.98 [M–C_2_HO_4_]^−^). This is the first mass spectrometric evidence of simultaneous eumelanin and pheomelanin use in a molluscan shell. Further investigations on the spatial distribution of these melanic pigments within the shell are needed to clarify their contribution to band and background colouration.

We could identify both eumelanin markers in one out of three individuals of the colourful marine gastropod *Clanculus pharaonius* (strawberry topshell) (Figs. 1 and 2). However, no obvious linkage of this sporadic finding to the shell phenotype was found (see Additional file 1)*.* Using HPLC–UV but without additional mass information, the eumelanin marker PTCA was recently also identified in another study of *Clanculus pharaonius* [21].

In the marine gastropod *Steromphala adriatica* (Fig. 1) we found an abundance of eumelanin markers (Fig. 2 and Table 2). This species is known to live in shallow waters in the Mediterranean Sea grazing on microfilm algae [49]. Melanin incorporation into the outer shell layers might therefore play a role in UV protection (similar as in the case of human skin [50]), habitat blending or shell strengthening as has been reported in other species [51], but further research is needed to functionally characterise this melanic pigment in *Steromphala adriatica.*

### The surprising absence of melanins in diverse pigmented mollusc shells

Surprisingly, no traces of melanin oxidation products were detected for many prominently patterned and brown coloured mollusc shells (Fig. 1, Table 1). This is especially surprising as brown and black colour patterns on bivalve and gastropod shells have generally been believed to be of melanic origin since the early studies of Comfort [7, 14, 16, 18]. Moreover, for some of the species we investigated the absence of melanin is in direct contrast with previous studies. For example, in a recent study [33] analysis of melanin oxidation products by HPLC with UV detection suggested that the brown valve of the bicoloured bivalve *Mizuhopecten yessoensis* (named *Patinopecten yessoensis* in Sun et al.) contains eumelanin and pheomelanin. However, in that study peak identification relied solely on retention times and no identification with mass data was used to verify those results. This can easily lead to the misidentification of melanin oxidation products (see [44]). Similarly, pigmentation present in the dark adductor scar of *Crassostrea gigas* was assumed to be eumelanin [30]. However, that result was based solely on measurements obtained by UV spectrophotometry and IR spectroscopy [30]. During sample preparation we observed acid solubility and fluorescence of pigments from the shell of *Crassostrea gigas*, possibly indicating porphyrin-like pigments known to be produced by the bivalves *Pinctada* spp. and *Pteria penguin* [52, 53].

The method we have used to detect melanin oxidation products was developed and adapted for challenging biological sample matrices such as molluscan shells and is highly sensitive (limit of detection ranging from 0.03 μg/mL to 0.10 μg/mL for UV detection, MS detection was even more sensitive) [44]. We also made additional efforts to detect melanins in the intensely brown coloured *Conus marmoreus* shell (for example grinding of the shell before dissolution and extended oxidation times) that were unsuccessful. We are therefore confident that in the indicated specimens melanin is genuinely absent (or exists in trace amounts inadequate to appreciably pigment the shell). This leads us to question what the prominent brown to black pigments are in shells where no melanin is detected. For some cases (e.g. *Lioconcha tigrina*) we observed that the geometric configuration of the pigmented pattern on the shell is preserved even after the calcium carbonate is dissolved in high molarity acid. This may suggest a stable macromolecular pigment. Unfortunately we were not able to identify other oxidation products in our samples that would indicate the chemical composition of these pigments. Further investigations are necessary to unravel their nature.

## Conclusions

We have found mass spectrometric evidence for melanins in three conchiferan classes: Cephalopoda, Gastropoda and Bivalvia. This is the first time melanin has been detected in a cephalopod shell (*Nautilus pompilius*). In the marine bivalve *Mytilus edulis* eumelanin is predominantly located in the periostracum layer relative to the calcified shell. For the first time both eumelanin and pheomelanin were detected in a mollusc shell (the terrestrial gastropod *Cepaea nemoralis*), however further study is needed to spatially localise the distribution of these pigments in this shell. Eumelanin markers could only be detected in one out of three *Clanculus pharaonius* individuals. In another marine gastropod (*Steromphala adriatica*) eumelanin was abundant. We could not detect melanin in a surprisingly large number of prominently patterned gastropod and bivalve shells. Further investigations are needed to identify the underlying pigmentation mechanism responsible for these complex geometric colourations.

## Material and methods

### Samples and standards

Shells from 13 different mollusc species were obtained either commercially or by donation from the Natural History Museum Vienna or private collectors for analysis (see Fig. 1 for images of samples used and Table 1 for previous literature and sample sources). For species previously reported to contain eumelanin in their shells (*Crassostrea gigas*, *Mizuhopecten yessoensis*, *Clanculus pharaonius* [21, 30, 33]) three replicates were analysed. For *Mizuhopecten yessoensis* the brown coloured left valve and for *Cepaea nemoralis* a morph with yellow background and multiple brown bands was analysed. For *Mytilus edulis* the periostracum was removed by scrubbing the shell with sand for one shell valve, while the other valve remained intact. As *Steromphala adriatica* are very small, seven shells were combined into one sample. Samples contained 0.9 to 2.2 g of shell material each. For *Lioconcha ornata* 0.5 g of shell material was available. Note that shells displaying multiple colours were not fragmented or sorted into colour groups. For the *Crassostrea gigas* sample material was taken from the internal shell surface in the region of the adductor scar. Care was taken to exclude pigmented material from the outer shell layers in this case. For comparison, standards of the melanin oxidation products PDCA, PTCA, TDCA and TTCA kindly provided by Prof. Ito were used.

### Sample preparation, melanin oxidation and HPLC–UV–MS analysis

Samples were processed as previously described [44]. In brief, shells were cleaned in deionized water, dried and weighted, and then dissolved in 6 M HCl. Residues were washed with water and were treated with proteinase K in 1 M Tris-HCl buffer at 37 °C for 2 h. Pigmented residues were treated with alkaline oxidation via H_2_O_2_ [41]: Oxidation reactions for each sample were carried out for 20 h at 25 °C under vigorous shaking using 100 μL H_2_O, 375 μL 1 M K_2_CO_3_ and 25 μL 30% H_2_O_2_ as reactants. The remaining H_2_O_2_ was decomposed by the addition of 50 μL 10% Na_2_SO_3_ and the mixture was acidified with 140 μL 6 M HCl. The solutions were then centrifuged and supernatants were transferred to fresh tubes.

Samples were treated by solid-phase extraction (Phenomenex Strata-X Polymeric Reversed Phase columns, 33 μm). Columns were conditioned with methanol (MeOH) followed by H_2_O. Shell extracts were loaded onto the columns and washed with 0.3% formic acid. Columns were dried and elution was carried out with MeOH followed by ethyl acetate. Solvents were removed under constant nitrogen stream at 40 °C and samples were dissolved in 200 μL H_2_O. Unless otherwise indicated samples were directly analysed following solid-phase extraction.

Measurements were carried out on a Thermo Fisher Scientific HPLC–MS system consisting of an Accela HPLC with a Finnigan Surveyor PDA Detector coupled to an LTQ Orbitrap XL mass spectrometer equipped with an electrospray ionisation (ESI) source. Separation was performed on a Phenomenex Gemini C18 column (250 × 2 mm, 5 μm). The mobile phase was 0.3% formic acid in H_2_O:MeOH (80:20). Analyses were performed at 45 °C at a flow rate of 0.2 ml/min. UV data were recorded in the range of 200–400 nm. Mass spectra were acquired in negative-ion mode over an *m*/*z* range of 120–220. Identification of melanin oxidation products were based on exact mass data and retention times. Quantitation was carried out by HPLC–UV in the range of 250–290 nm using external calibration with melanin oxidation product standards. Evaluation of HPLC–UV–MS data was performed using Thermo Xcalibur version 2.2.

## Supplementary information

**Additional file 1. **The three replicate samples of *Clanculus pharaonius* analysed in this study.

## Data Availability

The raw HPLC–UV–MS data generated by the work described in this paper is available from the Dryad repository (10.5061/dryad.h70rxwddx).

## Abbreviations

DOPA: L-3,4-dihydroxyphenylalanine
HCl: Hydrochloric acid
HPLC–MS: High-performance liquid chromatography with mass spectrometric detection
HPLC–UV: High-performance liquid chromatography with UV detection
PDCA: Pyrrole-2,3-dicarboxylic acid
PTCA: Pyrrole-2,3,5-tricarboxylic acid
TDCA: Thiazole-4,5-dicarboxylic acid
TTCA: Thiazole-2,4,5-tricarboxylic acid
