# Supplementary material for: Eumelanin and pheomelanin pigmentation in mollusc shells may be less common than expected: insights from mass spectrometry
Source: Front Zool. 2019 Dec 23;16:47. doi: 10.1186/s12983-019-0346-5 (PMC6929474; doi:10.1186/s12983-019-0346-5)
Supplement: Supplementary file 1 — Additional file 1. The three replicate samples of Clanculus pharaonius analysed in this study. [file 12983_2019_346_MOESM1_ESM.pdf]

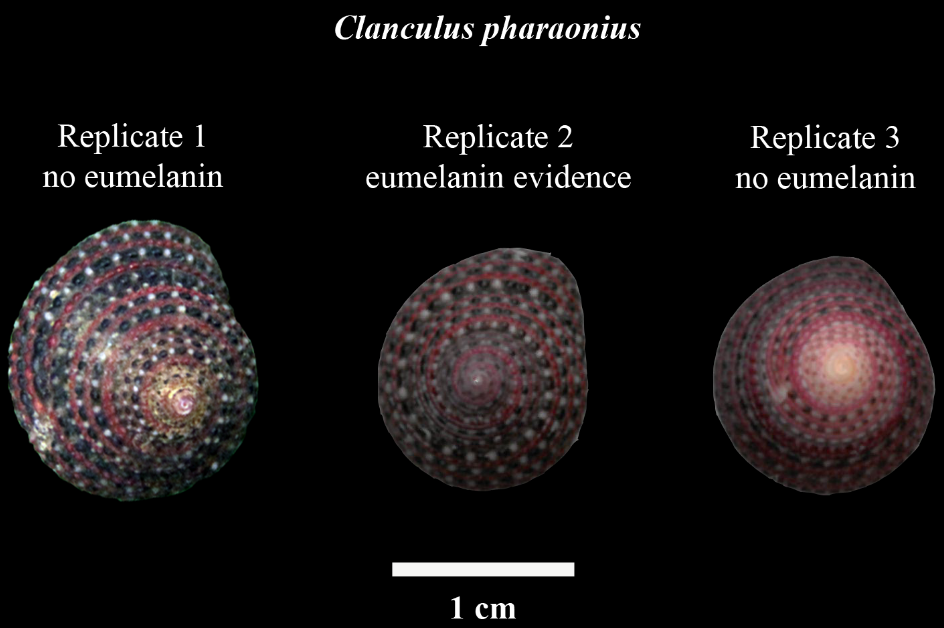
Clanculus pharaonius
Replicate 1
no eumelanin
Replicate 2
eumelanin evidence
Replicate 3
no eumelanin
1 cm
